# Impact of radiation therapy on survival in patients with triple-negative breast cancer

**DOI:** 10.3892/ol.2013.1700

**Published:** 2013-11-22

**Authors:** LAUREN T. STEWARD, FENG GAO, MARIE A. TAYLOR, JULIE A. MARGENTHALER

**Affiliations:** 1 Department of Surgery, Washington University School of Medicine, St. Louis, MO 63110, USA; 2 Division of Biostatistics, Washington University School of Medicine, St. Louis, MO 63110, USA; 3 Department of Radiation Oncology, Washington University School of Medicine, St. Louis, MO 63110, USA

**Keywords:** triple-negative breast cancer, radiation therapy, survival

## Abstract

Triple-negative breast cancer (TNBC) has a poorer prognosis compared with other sub-groups. In the current study, survival associated with locoregional treatment of females with TNBC was investigated. Specifically, 468 patients with stage I–III TNBC treated between 2002 and 2009 were identified. Data included patient and tumor characteristics, treatment received and survival. Data were compared using χ^2^ and Fisher’s exact tests, as well as MANOVA. Kaplan-Meier curves were generated. The study cohort had a mean age of 54±13 years old with a mean follow-up period of 51±21 months. Of 468 patients, 249 (53%) underwent lumpectomy, 63 (14%) underwent simple mastectomy (SM) and 156 (33%) underwent modified radical mastectomy (MRM). Overall, 263 (56%) received adjuvant radiation, including 178/249 (71%) following lumpectomy, 13/63 (21%) following SM and 72/156 (46%) following MRM (P<0.0001). Following control for potential confounders in univariate tests, adjuvant radiation was associated with improved overall survival in the total cohort (HR, 0.46; 95% CI, 0.31–0.68; P=0.0001). When comparing survival by surgical type, receipt of adjuvant radiation significantly improved survival in the lumpectomy group (HR, 0.30; 95% CI, 0.16–0.58; P=0.0004), but was not associated with improved survival in the SM group (HR, 0.38; 95% CI, 0.05–3.04; P=0.36) or in the MRM group (HR, 0.79; 95% CI, 0.46–1.34; P=0.38). The survival benefit of adjuvant radiation in these TNBC patients is attributed to those undergoing breast-conserving therapy. There was no benefit in either mastectomy group. These data warrant validation from prospective trials, in order to develop tailored locoregional treatment for patients with TNBC.

## Introduction

Triple-negative breast cancer (TNBC) accounts for 15–20% of all breast cancers in the USA (1–3). Treatment for TNBC [tumors that are estrogen receptor (ER)-negative, progesterone receptor (PR)-negative and human epidermal growth factor receptor 2 (HER2) non-amplified] continues to be a challenge due to the fact that it is, by definition, insensitive to the hormonal therapies and trastuzumab that have been developed to treat other types of breast cancer. These tumors appear to be exquisitely sensitive to chemotherapy with reported complete pathological response in 21–31% of TNBC tumors in patients treated with neoadjuvant chemotherapy (4,5). Despite this, these tumors are considered aggressive and have shorter intervals for locoregional recurrence, distant metastasis and disease-free survival (1,6–8). Gene expression profiling has resulted in the classification of breast cancer into five molecular subtypes: Luminal A (ER+, PR+ and Her2−), luminal B (ER+, PR+ and Her2+), basal-like (ER−, PR− and Her2−; triple-negative) Her2-enriched (ER−/PR−/Her2+) and the normal breast-like subtype. Recent studies have focused on whether molecular subtype is indicative of prognosis and response to treatment, but the data on directed-associated treatment is still in its infancy (6–8). As a result, chemotherapy continues to be the mainstay of treatment for TNBC breast cancers. However, recently, there has been interest in determining whether radiation therapy provides any additional benefit to patients with TNBC tumors, regardless of initial surgical management.

There are currently no specialized guidelines for the treatment of TNBC. In general, radiation therapy is indicated for all patients with invasive carcinoma of the breast under the following conditions: i) received breast-conserving therapy (BCT); ii) underwent mastectomy with tumor >5 cm or with positive margins; or iii) underwent mastectomy with positive axillary nodes. Recently, however, there have been several studies that have aimed to determine the outcomes of patients with TNBC tumors who received radiation therapy, in comparison to those who did not. The results of these studies appear to indicate that patients with TNBC tumors who received radiation therapy had decreased risk of locoregional recurrence and increased overall survival in comparison to those that did not receive radiation therapy (9,10). As a result, the present study was performed in order to determine whether similar results were observed in our study population.

## Materials and methods

### Study design

Approval from the institutional review board of Washington University School of Medicine (St. Louis, MO, USA) was obtained prior to the initiation of this study. It was determined that written consent from patients was not required given the retrospective nature of the study. We retrospectively identified 493 patients from our prospectively maintained database with a diagnosis of stage I–III TNBC who were treated between January 1, 2002 and December 31, 2009. Of these, 25 patients were diagnosed with stage IV breast cancer at the time of diagnosis and subsequently excluded from the analysis. As a result, 468 patients were included in the total study population. Patients were determined to have a TNBC based on immunohistochemical methods. A designation of receptor negative status was conducted based on having <1% stained cells. Fluorescence *in situ* hybridization was used to confirm HER-2/neu status if immunohistochemistry detected 2+ staining. Patients were subsequently divided based on whether they underwent lumpectomy or BCT versus simple mastectomy (SM) versus modified radical mastectomy (MRM). Data collected included patient and tumor characteristics; surgical, systemic and radiation treatment received; and breast cancer-specific survival.

### Statistical analysis

The primary outcome was overall survival (OS), which was defined as time from the date of treatment initiation to the date of mortality due to any cause. Survivors were censored at the date of last contact. The distributions of patient and clinical characteristics (including age, ethnicity, nodal status, tumor grade and size, receipt of chemotherapy and type of surgery) by the status of radiotherapy were compared using χ^2^ or Fisher’s exact tests, as appropriate. Survival curves by radiotherapy status were estimated using the Kaplan-Meier product-limit method and compared by the log-rank test. Univariate Cox proportional hazard models were fit to identify factors significantly associated with OS. For those factors with P<0.15 in the univariate analyses, a multivariate Cox model was constructed using a backward selection procedure to assess whether the receipt of radiotherapy was an independent predictor of survival. Two-way interaction terms between radiotherapy and other factors in the multivariate Cox model were also assessed. All analyses were two-sided and P<0.05 was considered to indicate a statistically significant difference. Statistical analyses were performed using SAS (SAS Institute, Cary, NC, USA).

## Results

During the study period, between January 2002 and December 2009, 468 patients with stage I–III TNBC were identified. Of 468 patients, 249 (53%) underwent lumpectomy, 63 (14%) underwent simple mastectomy and 156 (33%) underwent modified radical mastectomy. The mean age of the study population was 54±13 years old with a mean follow-up period of 51±21 months. The patient and tumor characteristics of the study population are described in Table I.

Overall, 263 (56%) received adjuvant radiation therapy, including 178/249 (71%) following lumpectomy, 13/63 (21%) following SM and 72/156 (46%) following MRM (P<0.0001), as listed in Table II. Of the 263 patients that received adjuvant radiation therapy, information regarding their treatment regimen was only available for 152 patients (57.8%). For these patients, the mean initial radiation dose was 5,137±938 cGy with a range of 2,000–10,240 cGy and median of 5,000 cGy. Of these patients, 84 (55.3%) went on to receive an additional boost of radiation with a mean of 1,292±629 cGy, median of 1,000 cGy and range of 1,000–6,400 cGy. Factors predictive of receipt of adjuvant radiation included type of surgical therapy received (lumpectomy vs. SM and MRM), increasing tumor size and positive nodal status (P<0.05 for each). The groups did not differ with regard to age, ethnicity, tumor size or nuclear grade (Table III).

**Figure 1:**
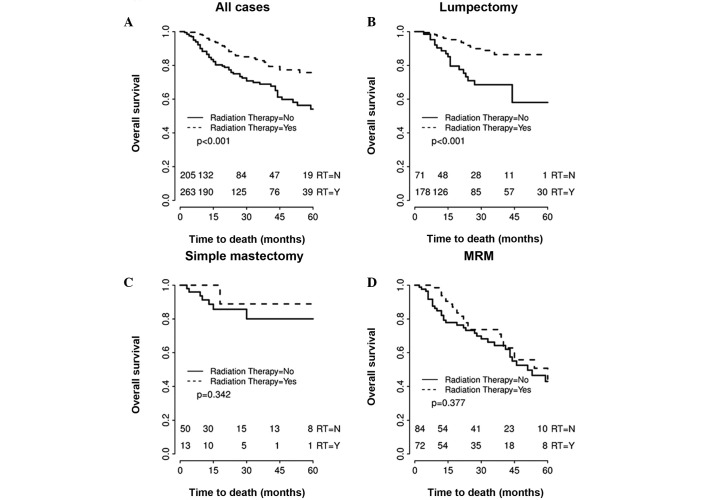
Kaplan-Meier survival curves for 468 patients with triple-negative breast cancer according to receipt of adjuvant radiation therapy versus no radiation therapy (RT). (A) Entire cohort of 468 patients. (B) Patients who underwent lumpectomy (n=249). (C) Patients who underwent simple mastectomy (n=63). (D) Patients who underwent modified radical mastectomy (MRM) (n=156).

In the total cohort, univariate analysis demonstrated that TNBC patients who underwent radiation therapy had significantly improved overall survival (HR, 0.462; 95% CI, 0.311–0.69; P=0.0001) compared with those who did not receive adjuvant radiation therapy. The overall four-year survival for patients who received adjuvant radiation therapy was 77.34 versus 59.8% in patients who did not receive adjuvant radiation therapy. Smaller tumor size (T1/T2), negative nodal status, receipt of systemic chemotherapy and receipt of adjuvant radiation therapy were all significantly associated with improved overall survival (P<0.05 for each). However, when comparing survival by surgical type, receipt of adjuvant radiation therapy significantly improved survival in the lumpectomy group (HR, 0.30; 95% CI, 0.16–0.58; P=0.001), but was not significantly associated with improved survival in the SM group (HR, 0.38; 95% CI, 0.05–3.04; P=0.34) or in the MRM group (HR, 0.77; 95% CI, 0.46–1.34; P=0.38). Fig. 1 illustrates the survival curves for all TNBC study patients, as well as the survival curves for patients treated by surgical intervention. Overall four-year survival for patients treated by surgical intervention was 78.9, 81.78 and 54.26% for lumpectomy, SM and MRM groups, respectively.

## Discussion

The majority of studies have shown that TNBC is a particularly aggressive form of breast cancer. These tumors tend to present in younger patients, at a larger size (>2 cm), with positive lymph nodes and with a higher mitotic index and grade (3,8). Patients with TNBC demonstrate poorer overall breast cancer-specific survival and shorter time to recurrence, including locoregional recurrence and distant metastasis (3,8,11,12). In addition, studies have also documented that the incidence of locoregional recurrence in TNBC patients peaks during years 1–4, but then sharply declines (8). Another study demonstrated that individuals with TNBC were more likely to have locoregional failure, in comparison to distant metastasis (11). As a result, the impetus for identifying the optimal locoregional treatment strategy for TNBC is of paramount importance. Yet, the connection between locoregional control and survival has yet to be elucidated for this sub-group of patients.

Currently, there are no specific guidelines for the management of TNBC. Systemic chemotherapy continues to be the mainstay of treatment, as the majority of TNBCs tend to be exquisitely sensitive to chemotherapy. However, how this systemic therapy specifically impacts locoregional control remains less clear. Radiation therapy is indicated for the majority of patients who undergo BCT and is also indicated for a sub-set of patients following mastectomy if high-risk features for locoregional recurrence exist, for example multiple positive lymph nodes, tumors >5 cm, presence of lymphovascular invasion or positive surgical margins. There are no tumor subtype-specific guidelines regarding adjuvant radiation therapy. Given that adjuvant radiation therapy is used for local control and TNBCs appear to have a higher incidence of locoregional recurrence, there has been recent interest in determining whether TNBC, a specific subtype of breast cancer, is likely to benefit from radiation therapy regardless of surgical intervention. Therefore, several retrospective studies have analyzed the role of radiation therapy in TNBC, but their findings are conflicting (11–14).

Dragun *et al* found that there was no difference in progression-free and locoregional-free survival in TNBC patients with or without radiation therapy during years 1–3. However, the radiation group had a higher probability of locoregional-free survival after three years (13). Abdulkarim *et al* found that T1-2N0 TNBC patients treated with MRM without RT had a significantly increased risk of locoregional recurrence in comparison with those treated with BCT, but there was no difference in overall survival (9). Wang *et al* completed a randomized trial comparing adjuvant chemotherapy versus adjuvant chemotherapy and radiation in stage I and II TNBCs who underwent modified radical mastectomy. The authors found improved recurrence-free and overall survival in patients who received combined therapy in comparison to those who only received chemotherapy (10). While the data appears to be inconsistent with respect to whether radiation decreases locoregional recurrence, there is even less clear evidence of the effect of radiation therapy on survival.

The current study has several limitations. Firstly, this is a retrospective study and, therefore, patients were not randomized to receipt of radiation therapy. There are specific guidelines regarding indications for adjuvant radiation therapy, but it is clear that adherence to these guidelines may not always occur and the reasons for this are unclear retrospectively. For example, it is noteworthy that of the 249 patients who underwent BCT, 71 patients (28.5%) did not receive any radiation therapy. It would be interesting to know if there were clinicopathological versus social factors that affected why these patients did not receive radiation. Another weakness of the study is the lack of consistency amongst the treatment regimens, including the radiation therapy regimen and whether or not patients received chemotherapy. The median radiation dose was 5,000 cGy and ~50% of those patients received an additional median boost of 1,000 cGy. These are fairly standard regimens, but the regimens did vary, reflecting the heterogeneity of patients that receive some or all of their care at our single institution. In future prospective studies, a more standardized radiation therapy must be outlined.

Although the retrospective nature of this study is a potential limitation, we propose that it represents one of the largest analyses of the impact of adjuvant radiation therapy in patients with TNBC, with the primary goal of determining the impact of radiation therapy on overall survival, rather than on locoregional recurrence. The study indicates that, while overall survival of patients with TNBC improved with radiation therapy, this improvement was attributed to those patients who underwent BCT. There was no difference in the overall survival of patients who underwent either form of mastectomy according to receipt of radiation therapy. These observations corroborate those documented by Kyndi *et al* in their study of high-risk patients who underwent MRM (14). The authors also found no survival benefit for post-mastectomy radiation in patients with TNBC.

Radiation therapy is not innocuous, nor is it without cost. Determining whether certain sub-groups of patients with TNBC may be able to forego adjuvant radiation therapy is of significant clinical interest.

## Figures and Tables

**Table I:** Patient and tumor characteristics of 468 patients with triple-negative breast cancer treated between 2002 and 2009.

Characteristic	n (%)
Age, years	
<50	193 (41.2)
≥50	275 (58.8)
Ethnicity	
Caucasian	287 (61.3)
African American	172 (36.8)
Other	9 (1.9)
Clinical T stage	
T1	166 (35.5)
T2	176 (37.6)
T3	37 (7.9)
T4	30 (6.4)
Unknown	59 (12.6)
Histology	
Invasive ductal	386 (82.5)
Invasive lobular	9 (1.9)
Mixed/other	73 (15.6)
Nuclear grade	
1	6 (1.3)
2	55 (11.8)
3	391 (83.6)
Unknown	16 (3.4)
Node status	
N0	295 (63.0)
N1	100 (21.4)
N2	12 (2.6)
N3	16 (3.4)
Unknown	45 (9.6)
Stage	
1	149 (31.8)
2a	142 (30.3)
2b	54 (11.5)
3	66 (14.1)
Unknown	57 (12.2)

**Table II:** Locoregional treatment of 468 patients with triple-negative breast cancer.

Type of surgery	Received radiation, n (%)	No radiation, n (%)
Breast-conserving therapy	178 (71.5)	71 (28.5)
Simple mastectomy	13 (20.6)	50 (79.4)
Modified radical mastectomy	72 (46.2)	84 (53.8)

**Table III:** Correlation between patient and tumor characteristics and receipt of radiation therapy in 468 patients with triple-negative breast cancer.

Characteristic	Radiation, n (%)	No radiation, n (%)	P-value
Age, years			
<50	83 (40.5)	110 (41.8)	NS
≥50	122 (59.5)	153 (58.2)	
Ethnicity			
Caucasian	125 (61.0)	162 (61.6)	NS
African American	77 (37.6)	95 (36.1)	
Other	3 (1.5)	6 (2.3)	
Clinical T stage			
T1	72 (35.1)	94 (35.7)	NS
T2	75 (36.6)	101 (38.4)	
T3	14 (6.8)	23 (8.8)	
T4	11 (5.4)	19 (7.2)	
Unknown	33 (16.1)	26 (9.9)	
Nuclear grade			
1	2 (1.0)	4 (1.5)	NS
2	22 (10.7)	33 (12.6)	
3	170 (82.9)	291 (84.0)	
Unknown	11 (5.4)	5 (1.9)	
Node status			
N0	141 (68.8)	154 (58.6)	0.0047
N1	37 (18.1)	63 (24.0)	
N2	0 (0)	12 (4.6)	
N3	7 (3.4)	9 (3.4)	
Unknown	20 (9.8)	25 (9.5)	
Chemotherapy			
Adjuvant	106 (51.7)	128 (48.7)	<0.0001
Neoadjuvant	45 (22.0)	106 (40.3)	
Unknown	54 (26.3)	22 (11.0)	
